# Pathogenicity and Transmissibility of Clade 2.3.4.4h H5N6 Avian Influenza Viruses in Mammals

**DOI:** 10.3390/ani12223079

**Published:** 2022-11-09

**Authors:** Cheng Zhang, Huan Cui, Chunmao Zhang, Kui Zhao, Yunyi Kong, Ligong Chen, Shishan Dong, Zhaoliang Chen, Jie Pu, Lei Zhang, Zhendong Guo, Juxiang Liu

**Affiliations:** 1 College of Veterinary Medicine, Hebei Agricultural University, Baoding 071000, China; 2 Changchun Veterinary Research Institute, Chinese Academy of Agriculture Sciences, Changchun 130122, China; 3 College of Animal Medicine, Jilin University, Changchun 130062, China

**Keywords:** chickens, avian influenza, H5N6 subtype, pathogenicity, transmissibility

## Abstract

**Simple Summary:**

In the past decade, the spread of H5N6 avian influenza viruses (AIVs) in birds and infection in humans has attracted increasing global attention, and these viruses have the potential to become a pandemic threat to global health. In this study, the HA genes of the HB1907 and HB1905 AIVs were clustered in the 2.3.4.4h clade, and the HA genes of both strains exhibited highly pathogenic avian influenza virus (HPAIV) characteristics. The HB1905 strain in this study has a binding preference for avian-like (α-2,3) receptors only, whereas the HB1907 strain has a binding preference for both avian-like (α-2,3) and human-like (α-2,6) receptors. Compared with the HB1905 strain, the HB1907 strain showed better replication ability in MDCK cells in the early stage of infection. At the same time, the HB1907 strain showed advantages in the pathogenicity of mice and the transmission ability of direct contact between guinea pigs. These results further suggest that epidemiological surveillance and the related studies of H5N6 AIVs are essential for public health safety and the healthy and sustainable development of the livestock industry.

**Abstract:**

Avian influenza viruses (AIVs) have the potential for cross-species transmission and pandemics. In recent years, clade 2.3.4.4 H5N6 AIVs are prevalent in domestic poultry, posing a threat to the domestic poultry industry and public health. In this study, two strains of H5N6 AIVs were isolated from chickens in Hebei, China, in 2019: A/chicken/Hebei/HB1907/2019(H5N6) and A/chicken/Hebei/HB1905/2019(H5N6). Phylogenetic analysis showed that both viral HA genes clustered in the 2.3.4.4h clade. Receptor binding analysis showed that the HB1905 strain preferentially binds to α-2,3-linked sialic acid (SA) receptors, while the HB1907 strain preferentially binds to α-2,3- and α-2,6-linked sialic acid (SA) receptors. During early infection, the HB1907 strain is highly replicable in MDCK cells, more so than the HB1905 strain. Pathogenicity assays in mice showed that both viruses could replicate in the lungs without prior adaptation, with HB1907 being more highly pathogenic in mice than the HB1905 strain. Significantly, both the HB1905 and HB1907 strains can be transmitted through direct contact among guinea pigs, but the transmission efficiency of the HB1907 strain through contact between guinea pigs is much greater than that of the HB1905 strain. These results strengthen the need for ongoing surveillance and early warning of H5N6 AIVs in poultry.

## 1. Introduction

Highly pathogenic avian influenza (HPAI) H5N1 was first isolated in Guangdong Province, China, and subsequently reported worldwide [1,2]. The genetic evolution of H5 subtype avian influenza viruses (AIVs) has led to the differentiation and production of 10 different viral clades (0–9) and several subclades [3,4]. In 2014, a new clade of H5Nx AIVs (2.3.4.4) emerged with neuraminidase (NA) subtypes, including N2, N3, N6 and N8 [5]. H5N6 AIVs of the 2.3.4.4a strain were first reported in poultry in Laos in 2013, followed by outbreaks in China, South Korea and elsewhere [6]. The novel reassorted H5N6 AIVs of the 2.3.4.4 clade have been subject to increasing interest and concern due to their rapid evolution and global spread in recent years. Many birds, including wild waterfowl and poultry, are susceptible to the 2.3.4.4 clade AIVs. Over 33 million birds have been culled worldwide, causing significant economic losses to the poultry industry [3,4,7]. HPAI H5N6 virus of the 2.3.4.4 clade has also reportedly been detected in cats, pigs and humans [7,8,9]. These recurrent outbreaks of H5N6 AIVs pose an ongoing potential pandemic threat worldwide.

Wild birds and domestic poultry serve as reservoirs of H5N6 AIVs and can transmit the virus to humans. The first human influenza H5N6 AIV case was reported in Hunan Province, China, in 2014, and in the same year, the first death was reported in Sichuan Province, China [10,11]. At present, 65 cases of human infection with H5N6 AIVs, have been reported, with a mortality rate as high as 55% [12]. Some of the patients had a history of contact with infected poultry, raising concerns about its pandemic potential [13]. In addition, H5N6 AIVs can reassort with other subtypes (H3, H6, H7, H9 and H10) of AIV and generate a variety of novel variants [14,15,16]. In recent years, the 2.3.4.4h clade has been shown to be notably dominant in poultry in China; thus, its pathogenic and transmissible ability in mammals needs to be further studied. The latest study of swan-origin H5N6 AIVs isolated in 2020 and a study of H5N6 AIVs isolated from wild duck feces in 2022 both showed that the clade 2.3.4.4h H5N6 AIVs could infect mammals without adaptation and that they have certain pathogenicity [6,17]. Therefore, extensive surveillance of both wild birds and poultry is necessary to further elucidate the ability of the H5N6 AIV 2.3.4.4h clade to infect mammals, as well as to assess the potential threat to public health and livestock development from poultry sources.

In this study, two H5N6 AIVs were isolated in 2019 from chickens in Hebei Province: A/chicken/Hebei/HB1907/2019 (H5N6) and A/chicken/Hebei/HB1905/2019 (H5N6). In this study, we systematically analyzed and evaluated the receptor-binding properties, cell proliferation capacity, pathogenicity and transmissibility of these two H5N6 AIVs in mammals. These results expand our understanding of the pathogenicity and transmissibility of H5N6 AIVs and provide basic data and assistance for influenza pandemic preparedness.

## 2. Materials and Methods

### 2.1. Viruses

A/chicken/Hebei/HB1905/2019(H5N6) (abbreviated as HB1905) (GenBank: MZ618922–MZ618924) and A/chicken/Hebei/HB1907/2019(H5N6) (abbreviated as HB1907) (GenBank: MZ618947–MZ618949) were isolated from two free-range broiler farms in Hebei Province, China, in March 2019. The sampling design covered the entire areas of the two free-range broiler farms [18]. We collected samples from all diseased broilers [19]. The clinical sample collection and transportation were performed according to the Chinese Guidelines for the Technical Specifications for the Collection, Preservation and Transport of Highly Pathogenic Avian Influenza Samples. Typical influenza symptoms were observed in infected broilers, including clinical symptoms of neurological disease and diarrhea, and these clinical findings were consistent with previous reports [18,20]. All infected broilers died within 24 h after onset, and autopsy showed gastrointestinal bleeding, severe pneumonia and encephalitis. The incidence rate of chicken farms with the HB1905 strain isolated was as high as 80% (224/279), and the incidence rate of chicken farms with the HB1907 strain isolated was 95% (832/875). We collected oropharyngeal swabs and anal swabs from diseased broilers to obtain isolates. In brief, swabs were collected in 1 mL of phosphate-buffered saline (PBS). The supernatant was filtered using a 0.22 μm filter and inoculated into specific-pathogen-free (SPF) chicken embryos (Beijing Boehringer Ingelheim Viton Biotechnology Co., Ltd., Beijing, China). The virus was isolated and purified from SPF chicken embryos. Following incubation at 37 °C for 48 h, allantoic fluid was harvested and stored at −80 °C. All viruses were then passed three times by limiting dilution in SPF chicken embryos. Nucleic acid was extracted from allantoic fluid, and the detection of viral RNA was performed with real-time RT-PCR assay, as previously reported [21]. The results supported H5 positivity.

### 2.2. Viral Genome Sequencing and Analysis

The QIAamp Viral RNA Mini Kit (Qiagen, Germantown, MD, USA) was used to extract viral genomic RNA from allantoic fluid according to the manufacturer’s instructions. The PrimeScript™ RT Reagent Kit with gDNA Eraser (TaKaRa, Dalian, China) was used to transcribe viral genomic RNA into cDNA. PCR amplification was performed using primers specific to AIV as previously reported [22]. PCR products were purified using the TaKaRa MiniBEST DNA Fragment Kit Ver.4.0 (TaKaRa, Dalian, China). A BigDye™ Terminator V3.1 cycle sequencing kit (Applied Biosystems, Foster City, CA, USA) was used for sequencing. The SEQMAN program was used to analyze sequencing data (DNASTAR, Madison, WI, USA). From NCBI GenBank, reference sequences for the HA, NA and PB2 genes were retrieved. With Cluster W, the downloaded sequences were aligned and compared to the strains used in this work. The MEGA 7.0.21 program (Sinauer Associates, Inc., Sunderland, MA, USA) was used to perform a phylogenetic analysis based on the maximum likelihood (ML) with a bootstrap value of 1000. Figtree (v1.4.2, http://tree.bio.ed.ac.uk/software/figtree/) (accessed on 26 June 2022). was used to visualize the phylogenetic tree.

### 2.3. Receptor Binding Assay

The receptor-binding assays were conducted as described previously [23]. Briefly, HA assays were used to determine the receptor-binding preferences of the viruses using four different types of red blood cells (RBCs): chicken red blood cells (cRBCs) (Solarbio, Beijing, China, S9454) containing α-2,3 and α-2,6-linked sialic acid (SA) receptors; sheep red blood cells (sRBCs) (Solarbio, Beijing, China, TX0030) containing α-2,3-linked SA receptors; cRBCs treated with Takara α-2,3-Sialidase(Takara, Dalian, China), which only has α-2,6-linked SA receptors; and cRBCs treated with Vibrio cholerae NA (VCNA; Roche), which has no receptors. A/chicken/Hebei/HB777/2006 (H5N1) and A/California/04/2009 (H1N1) were used as controls. Next, 50 μL of virus was added and serially diluted in PBS in 96-well plates. Finally, different 1% RBC suspensions of 50 μL were added, respectively. The titer was read after 30 min of incubation at 25 °C.

### 2.4. Cell Growth Curves

Madin-Darby canine kidney (MDCK) cells were used for growth evaluation according to a previous study [24,25]. The two strains (HB1905 and HB1907) infected cells with an MOI of 0.01 (10^5^ cells). One hour after inoculation, the cells were washed twice with PBS, and fresh medium supplemented with 1 µg/mL tosyl phenylalanyl chloromethyl ketone (TPCK) and trypsin (Sigma Aldrich, St. Louis, MO, USA) was added. Cell supernatants were collected every 12-h post-infection (hpi) until the end of 60 hpi. All collected cell supernatants were inoculated into SPF chicken embryos, and EID_50_ values were calculated. The experiments were performed in triplicate.

### 2.5. Mouse Study

The method for the mouse study was performed according to previous studies [26,27]. Forty-eight 6 weeks old female BALB/c mice were purchased from Beijing Vital River Laboratory Animal Technology Co., Ltd. Fifteen BALB/c mice were randomly separated into three groups (n = 5 per group) and anesthetized with isoflurane. Two groups were inoculated intranasally with 50 μL of HB1905 or HB1907 at 10^6^ EID_50_. Control mice were inoculated intranasally with an equal volume of PBS. The weight loss and survival rates of the BALB/c mice in the three groups were monitored daily for 14 days. Mice that lost >20% of their body weight were euthanized. Thirty-three BALB/c mice were randomly separated into three groups (three for control and fifteen per group for inoculation). Two inoculated groups were anesthetized with isoflurane and intranasally inoculated with HB1905 or HB1907 virus at 10^6^ EID_50_, while the mice of the control group were intranasally inoculated with an equal volume of PBS. Three mice per inoculated group were euthanized at 1, 3, 5 and 7 days post-infection (dpi) to determine the viral load in the lungs. The lung samples were homogenized in 1 mL of PBS using a tissue lyser (Qiagen, Germany). Samples were clarified at 8000 rpm at 4 °C for 10 min. The above-clarified lung homogenates were inoculated into SPF chicken eggs, and the EID_50_ was determined by hemadsorption. At 5 dpi, the lungs of three BALB/c mice per group from the three groups were removed and fixed in formalin, embedded in paraffin, and stained with hematoxylin and eosin (H&E) for pathological examination.

### 2.6. Guinea Pig Study

Referring to previous studies [27,28,29], 18 female guinea pigs (300–350 g) were used in the transmission test. Three guinea pigs in each group were inoculated intranasally with HB1905 or HB1907 of 10^6^ EID_50_ at 200 μL. The next day, three uninfected and three infected guinea pigs were placed on the same side of the individually ventilated cages (IVC) for direct contact transmission. Three uninfected guinea pigs in each group were placed on the opposite side of the infected guinea pig cage at a contact distance of 5 cm, which allows air to flow in the cage but prevents contact between guinea pigs. In addition, the guinea pigs were housed in individually ventilated cages (IVC) and provided with sterile water, bedding and feed. The wind speed was set to 0.4 m/s [30]. Nasal washes were collected every two days, samples were inoculated into SPF chicken eggs for viral titer determination and sera were collected to determine seroconversion at 21 dpi.

### 2.7. Statistical Analysis

Statistically significant differences were determined using one-way analysis of variance (ANOVA) for parametric parameters and the Mann-Whitney test for nonparametric parameters with the GraphPad Prism 8 software (San Diego, CA, USA). All assays were performed in at least three independent experiments. The error bars represent the standard deviation.

## 3. Results

### 3.1. HB1907 Is Closely Related to the Human Strain

**Figure 1:**
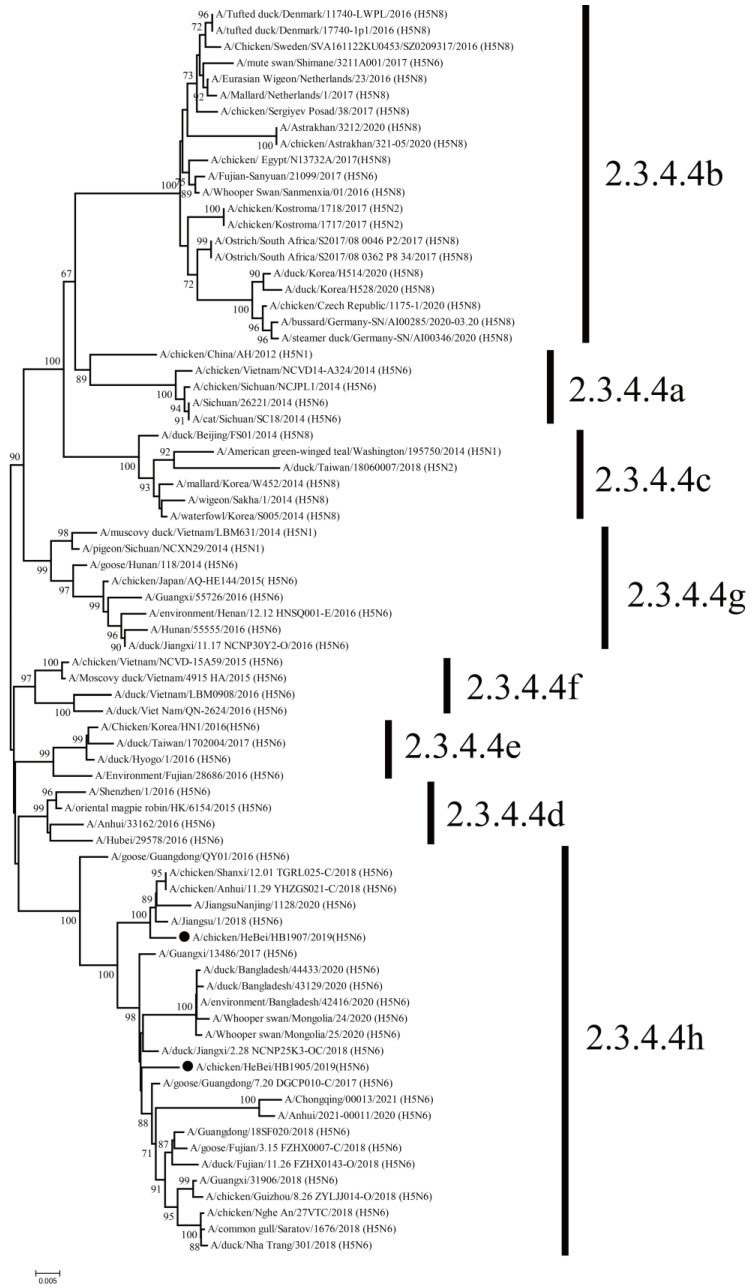
Phylogenetic tree of the HA genes of the H5N6 (HB1905 and HB1907) viruses. The tree was constructed by using MEGA 7.0.21 software using the maximum likelihood (ML) with a bootstrap value of 1000. Sequences in this study are marked with black circles.

**Figure 2:**
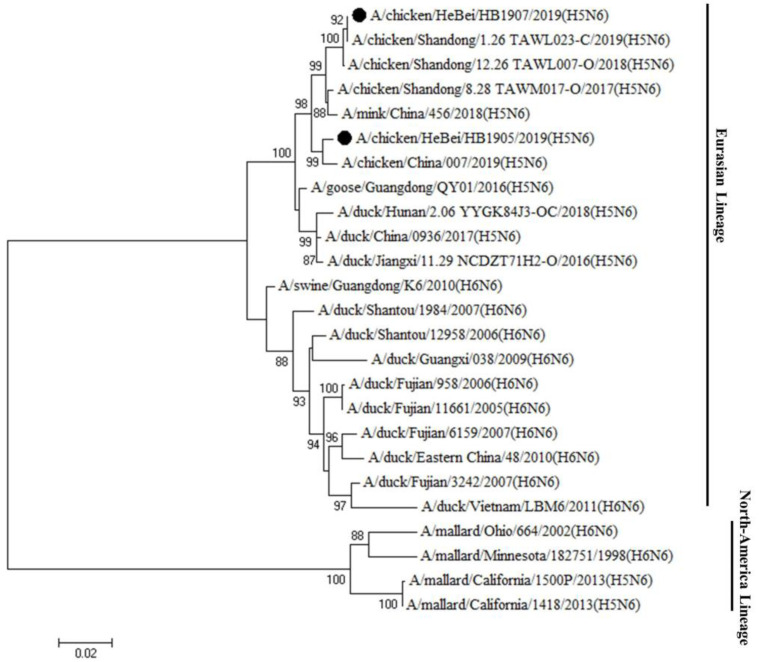
Phylogenetic tree of the NA genes of the H5N6 (HB1905 and HB1907) viruses. The tree was constructed by using MEGA 7.0.21 software using the maximum likelihood (ML) with a bootstrap value of 1000. Sequences in this study are marked with black circles.

Genetic evolution analysis showed that the HA genes of both strains were clustered in clade 2.3.4.4h (Figure 1). The cleavage site of the HA protein has been observed in multiple basic amino acids (RERRRKR↓G), which are associated with the characteristics of HPAIV. The HB1907 HA gene is closely related to human strains A/Jiangsu/1/2018 (H5N6) and A/Jiangsu Nanjing/1128/2020 (H5N6), with nucleotide similarities of 99.0% and 98.6%, respectively (Supplementary Table S2). The HA gene of HB1905 was closely related to the poultry strains A/duck/Jiangxi/2.28NCNP25K3-OC/2018 (H5N6) and A/Goose/Guangdong/7.20DGCP010-C/2017 (H5N6), with nucleotide similarities of 98.8% and 99.3%, respectively (Supplementary Table S2). Sequence analysis revealed 13 amino acid differences in the HA gene under the H3 numbering system between HB1905 and HB1907 (R44G; S77R; R125S; S128P; A145P; M166I; K173S; S187N; V188A; N193K; A218T; R227S; V531A). The NA genes of the two virus strains were clustered in the Eurasian lineage (Figure 2), with 11 missing amino acids in the NA stalk regions. There were 14 amino acid differences in the NA gene between the two strains (I26T; V39M; T43A; N46S; V76M; E81G; R130K; K250R; M262I; A266T; R286G; I342T; S345N; D386N). There were 24 amino acid differences in the PB2 gene between the two strains (R80K; S137N; M183L; I225V; K332R; I338V; R340K; V348L; L451I; I457V; V461I; N490S; Q508R; N559T; T598V; A613V; E627K; M636L; L648V; S661A; T676V; N680D; R699K; A731V). In Supplementary Table S1, the amino acid differences of the two strains of virus in this study are shown. The above differences in amino acid sites may be factors in the enhanced pathogenicity and transmission ability of strain HB1907.

### 3.2. Differences in Binding Affinity for Different Sialic Acid Receptors

**Figure 3:**
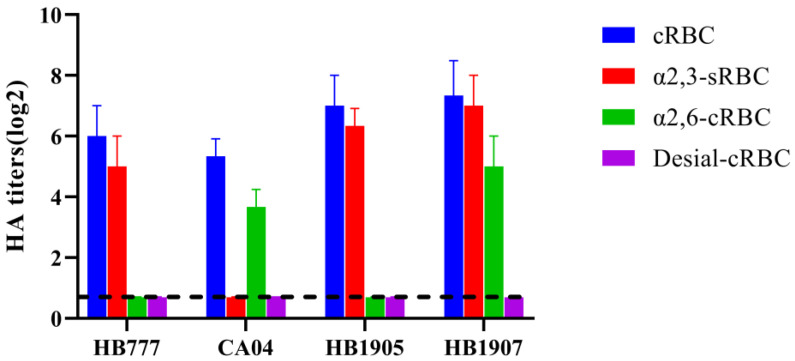
Receptor-binding specificity of H5N6 (HB1905 and HB1907) viruses. In the control group, HB777(H5N1) only preferred to bind avian-like (α-2,3) receptors, while CA04 (H1N1) only preferred to bind human-like (α-2,6) receptors. HB1905 prefers to bind avian-like (α-2,3) receptors. The HB1907 strain showed similar binding preferences with avian influenza virus receptors (α-2,3) and human influenza virus receptors (α-2,6). In each group, three separate experiments were carried out.

Influenza viruses invade host cells by binding the HA protein to SA receptors on the cell surface. Avian influenza virus preferentially binds to α-2,3 receptors, while human influenza virus preferentially binds to α-2,6 receptors [31,32]. As shown in Figure 3, A/chicken/Hebei/HB777/2006 (H5N1) could agglutinate only sRBCs containing α-2, 3-linked SA receptors, and A/California/04/2009 (H1N1) could agglutinate only cRBCs containing α-2,6-linked SA receptors. Both the HB1905 and HB1907 viruses could agglutinate untreated cRBCs containing α-2,3-linked and α-2,6-linked sialic acid receptors, but could not agglutinate VCNA-treated cRBCs containing no receptors. Both the HB1905 and HB1907 viruses could agglutinate the sRBCs containing α-2,3-linked sialic acid receptors, and only HB1907 could agglutinate the α-2,3-sialidase-treated cRBCs containing only α-2,6-linked sialic acid receptors. These results indicated that HB1905 prefers to bind only avian-like (α-2,3) receptors; HB1907 prefers to bind both avian-like (α-2,3) receptors and human-like (α-2,6) receptors. 

### 3.3. The HB1907 Virus Showed Better Replication in MDCK Cells

**Figure 4:**
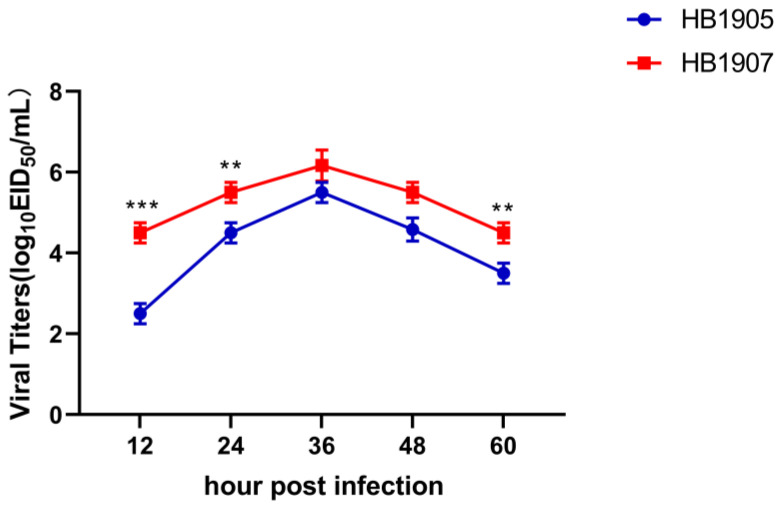
Virus titers at different time points in MDCK cells. The two strains (HB1905 and HB1907) infected cells with an MOI of 0.01 (10^5^ cells). At 12, 24, 36, 48 and 60 hpi, cell supernatants were collected and inoculated into SPF chicken embryos. The titer of the virus at different time points was determined by EID_50_. Three independent experiments were performed in each group (** *p* < 0.01, *** *p* < 0.001).

The growth curve in MDCK cell lines shows the viral replication abilities. The titers of the HB1905 and HB1907 viruses were highest at 36 hpi, 10^5.50^ EID_50_/mL, and 10^6.17^ EID_50_/mL, respectively (Figure 4). However, HB1907 was significantly higher than HB1905 at 12 hpi (*p* < 0.001) and 24 hpi (*p* < 0.01), which is an indication that the ability of the HB1907 virus to replicate was significantly higher than that of the HB1905 virus at the early stage of infection (Figure 4).

### 3.4. The HB1907 Strain Showed a Higher Pathogenicity

**Figure 5:**
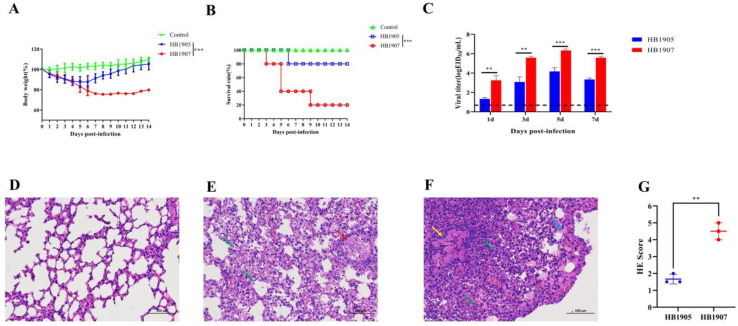
Pathogenicity in HB1905- and HB1907-infected mice. (**A**) Daily weight monitoring was performed for each group for 14 days. (**B**) The mortality rate of each group was recorded for 14 consecutive days. (**C**) Replication of HB1905 and HB1907 viruses in mouse lung. The dashed line represents the lower limit of virus detection. (**D**) Lung pathological sections of mice in the control group at 5 dpi. (**E**) Lung pathological sections of mice in the HB1905 group at 5 dpi. (**F**) Lung pathological sections of mice in the HB1907 group at 5 dpi. (**G**) Pathology scores in the lungs of infected BALB/c mice. Images were acquired using a ×20 magnification objective. Alveolar wall thickening (arrow green), lymphocyte infiltration (arrow red), epithelial cell necrosis (arrow yellow), inflammatory cell infiltration; (arrow blue), acidophilic protein-like exudation. Three independent experiments were performed in each group (** *p* < 0.01, *** *p* < 0.001).

There was a gradual decrease in weight in mice in the HB1905 group, before it began to rise at 5 dpi, while the weight of mice in the HB1907 group declined to approximately 76% at 9 dpi (Figure 5A). The survival rate of mice in the HB1905 group was 80%, but the survival rate of mice in the HB1907 group was 20%. Death occurred at 6 dpi in the HB1905-infected group and 3, 5 and 9 dpi in the HB1907-infected group (Figure 5B). Based on the data above, we conclude that HB1907 showed greater pathogenicity than HB1905 in mice (*p* < 0.001).

At 1, 3, 5 and 7 dpi, both viruses could be detected in the lungs of all the mice with titers ranging from 10^1.33^ to 10^6.33^ EID_50_/mL (Figure 5C). The viral titers of HB1907 were higher than those of HB1905 at 1, 3, 5 and 7 dpi (*p* < 0.01). The highest viral load was observed at 5 dpi. The titer of HB1907 was approximately 10^6.33^ EID_50_/mL at 5 dpi, which was 148-fold higher than that of HB1905.

As shown in Figure 5D–F, mice infected with HB1905 and HB1907 showed significant lung lesions. Pathological results were scored in each part of each lung: 0—no pathological changes; 1—lesion area ≤ 10%; 2—lesion area 10–50%; 3—lesion area ≥ 50%. When pulmonary edema and/or alveolar hemorrhage were scanned, the score was increased by 1 point. According to the above criteria, the pathogenicity of the HB1907 virus in mice was higher than that of the HB1905 virus (Figure 5G).

### 3.5. Direct Contact Transmission for HB1907 Virus Is Higher

**Figure 6:**
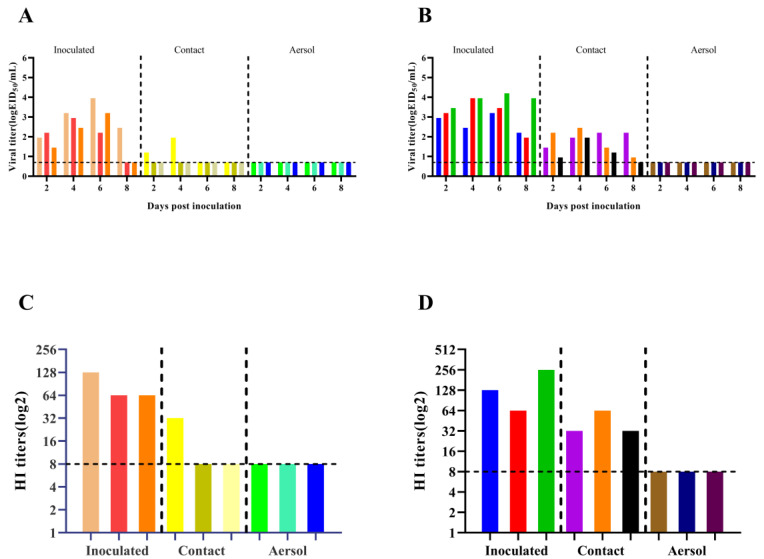
Transmission of H5N6 subtype avian influenza isolates (HB1905 and HB1907) in guinea pigs. (**A**) The *X*-axis shows guinea pigs infected with HB1905, exposed to direct contact, and transmitted by aerosols. On the *Y*-axis, influenza virus titers in guinea pig nasal rinse are shown. (**B**) On the *X*-axis, guinea pigs infected with HB1907, exposed to direct contact group, and transmitted by aerosols are shown. On the *Y*-axis, influenza virus titers in guinea pig nasal rinse are shown. (**C**) The *X*-axis shows guinea pigs infected with HB1905 group, with direct contact group, and with the aerosol transmission group, and the *Y*-axis shows HI antibody titers of different guinea pigs. (**D**) The *X*-axis shows guinea pigs infected with HB1907, directly exposed group and aerosol transmitted group, and the *Y*-axis shows HI antibody titers of different guinea pigs. Each color bar represents an individual guinea pig. The horizontal dashed line represents the lower limit of assay detection.

Guinea pig models have been widely used to assess the transmissibility of influenza viruses [33]. Guinea pig nasal wash and serum samples were harvested to detect virus titers and HI antibody titers. The transmission efficiency of the influenza virus was evaluated by detecting the virus-positive rate of guinea pig nasal wash in different transmission groups. As shown in Figure 6A, in the HB1905 contact group, only one of the three guinea pigs replicated the virus at 2 (10^1.20^ EID_50_/mL) and 4 (10^1.95^ EID_50_/mL) dpi with a direct-contact transmission efficiency of 33.3%. As shown in Figure 6B, the titers of 2 dpi nasal washes of guinea pigs in the HB1907 direct contact transmission group were 10^1.45^ EID_50_/mL, 10^2.20^ EID_50_/mL and 10^0.95^ EID_50_/mL, respectively. The titers of nasal washes at 4 dpi were 10^1.95^ EID_50_/mL, 10^2.45^ EID_50_/mL and 10^1.95^ EID_50_/mL, respectively. The titers of nasal washes at 6 dpi were 10^2.20^ EID_50_/mL, 10^1.45^ EID_50_/mL and 10^1.20^ EID_50_/mL, respectively. The three guinea pigs in the contact transmission group of HB1907 virus all shed detectable viruses with 100% efficiency for direct contraction. These results indicate that both HB1905 and HB1907 can be transmitted through direct contact, but the transmission efficiency of HB1907 was much higher than that of HB1905. Figure 6C–D show the HI antibody titers in guinea pig serum. Serum samples from the two strains of the virus-infected groups were positive, and serum samples from guinea pigs in the aerosol transmission group were negative. Serum samples from one of three guinea pigs that had direct contact with guinea pigs infected with HB1905 were positive, while serum samples from three guinea pigs that had direct contact with guinea pigs infected with HB1907 were all positive.

## 4. Discussion

HPAI H5N1 was first reported in 1996 on a goose farm in Guangdong, China, which caused a mortality rate of up to 40% [34]. Despite efforts to promote vaccines to control the H5 virus, unprecedented large-scale mutations of the virus have generated various genotypes or sublineages, causing huge economic losses in China [14,15,35,36]. Since 2013, an unprecedented outbreak of HPAI H5 clade 2.3.4.4 viruses has been reported among poultry and wild birds in China. The virus has multiple NA subtypes, including H5N2, H5N5, H5N6 and H5N8 strains, and the prevalence is increasing year by year [37,38]. Large poultry farms in northern China are not only potential victims of influenza outbreaks but also important sites for the natural mutation and cross-species spread of influenza viruses. Regular monitoring and the early warning of influenza virus on farms is very conducive to the prevention and control of new influenza outbreaks. 

Phylogenetic analysis of HA genes showed that the two H5N6 strains isolated in this study belong to the clade 2.3.4.4h H5N6 AIVs, which is consistent with the clade of H5N6 strains prevailing in China during 2018–2020 reported in previous studies [39]. At the same time, the HA proteins of the two strains in this study had multiple basic amino acids at the cleavage site (RERRRKR↓G), which is a characteristic of HPAIV [17,39,40]. These indicated that the viruses were capable of causing high death rates in poultry [6,41]. The HA gene of HB1907 was closely related to the human strains (A/Jiangsu/1/2018 (H5N6) and A/Jiangsu Nanjing/1128/2020 (H5N6)), and the HA gene of HB1905 was closely related to the poultry strains (A/duck/Jiangxi/2.28NCNP25K3-OC/2018 (H5N6) and A/Goose/Guangdong/7.20DGCP010-C/2017 (H5N6)). Previous studies have shown that avian influenza strains could be more transmissible and reproducible in mammals with higher homology to human strains [42,43]. The NA genes of the two strains of viruses were clustered in the Eurasian lineage, which is consistent with many H5N6 AIV subtypes isolated in recent years [37]. Given the rapid spread of the H5N6 AIVs in avian species and the ability to generate new recombinant strains, increasing attention should be given to prevent the reassortment of novel AIVs to infect humans or other animal species.

Receptor-binding preference is considered to be one of the important factors affecting the pathogenicity and transmissibility of influenza A virus [44]. The acquisition of the binding capacity of human-like (α-2,6) receptors by influenza viruses is an essential step in adaptation to human hosts [45]. The HB1905 strain in this study showed a binding preference for only avian-like (α-2,3) receptors, yet it replicated in mammals. The deletion of 11 amino acids in the NA stalk regions of the two strains in this study indicates that they may have certain adaptability and pathogenicity in mammals [46]. Interestingly, some new amino acid substitutions have also been detected in this study in addition to the mutations that led to the preference for avian-like receptors (Q226 and G228), which may be the reason for the above phenomena. In future research, it will be interesting to explore the functions of these unexplored amino acid mutations and their interactions with other amino acids. The HB1907 strain has a binding preference for both avian-like (α-2,3) and human-like (α-2,6) receptors. The HA gene of influenza A plays an important role in its receptor-binding preference. The receptor-binding sites of HB1905 and HB1907 both contained Q226 and G228, indicating an avian α-2,3-SA receptor binding preference [47,48]. However, the S128P mutation in HB1907 strain HA may enhance its adaptation and infectivity in mammals [49,50]. There are other amino acid sites in the HA gene that are different between the two strains, and whether they affect receptor binding preference remains to be further studied. 

The pathogenicity of HB1905 in mice was consistent with studies of swan-origin H5N6 AIV isolated in 2020 and H5N6 AIV isolated from wild duck feces in 2022 [6,17]. The pathogenicity of the HB1907 virus in mice was significantly higher than that of the HB1905 virus. Unlike HB1905, HB1907 has a 627K locus in the PB2 gene without mouse adaptation, which often indicates a potential risk of mammalian infection [51]. Whether other amino acid positions in the PB2 gene of both strains play a role in mammalian fitness and virulence remains to be further investigated. Given the rapid spread of H5N6 AIVs in avian species and the ability to generate new recombinant strains, increasing attention should be paid to the prevention of the reassortment of novel AIVs to infect humans or other animal species.

Previous studies have shown that clade 2.3.4.4 H5N6 AIVs can be transmitted by direct contact in guinea pigs or ferrets [26,52,53,54]. Our study showed that both HB1905 and HB1907 viruses could be transmitted by direct contact, and the transmission efficiency of the HB1907 virus through contact was significantly higher than that of the HB1905 virus. This indicates that our results are consistent with previous studies that the clade 2.3.4.4 H5N6 AIVs pose a potential pandemic threat. Multiple parameters, such as receptor-binding ability, replication ability, and virus survival characteristics under various environmental circumstances, may influence the potential of AIVs to transmit between mammals [55]. This study had several other limitations. First, although we carried out histological evaluations, we did not perform immunohistochemistry staining in the lung. Second, the data from mutation tracking and pathogenicity evaluation may not be sufficient to understand the possible pandemic risk of this naturally evolved H5N6 virus. Finally, this study is only a preliminary characterization of two chicken-origin H5N6 AIVs in mammalian models. Further mechanistic studies are needed in the future. Many other biotechnologies, including bioinformatics, synthetic biology and prediction models, will need to be employed as efficient tools in the future to determine more about the link between viruses and their hosts [56].

## 5. Conclusions

In conclusion, the two strains of H5N6 AIVs from the same branch in this study showed differences in mammalian models. We speculate that this difference may be caused by differences in amino acid mutation sites. Further studies are needed to investigate these findings, along with the biological roles of amino acid mutation sites. At the same time, the HB1907 virus in this study is highly replicated in MDCK cells, is highly pathogenic to mice, and has good direct contact transmission ability between guinea pigs. These results further suggest our long-term epidemiological surveillance of H5N6 AIVs and related research. It is beneficial to human public health security and the healthy and sustainable development of animal husbandry. Research in mammalian models broadens our knowledge of chicken-origin H5N6 AIV clade 2.3.4.4h.

## Data Availability

The study’s original contributions are included in the article; further inquiries can be directed to the corresponding authors.

## Supplementary Materials

The following supporting information can be downloaded at: https://www.mdpi.com/article/10.3390/ani12223079/s1, Table S1: Amino acid differences between two H5N6 influenza viruses; Table S2: Nucleotide similarity of HA gene of two H5N6 isolates.
