# Supplementary material for: Pathogenicity and Transmissibility of Clade 2.3.4.4h H5N6 Avian Influenza Viruses in Mammals
Source: Animals (Basel). 2022 Nov 9;12(22):3079. doi: 10.3390/ani12223079 (PMC9686590; doi:10.3390/ani12223079)
Supplement: Supplementary file 1 [file animals-12-03079-s001.zip › animals-1909757-supplementary.pdf]

animals

MDPI

Article

Pathogenicity and Transmissibility of Clade 2.3.4.4h H5N6 Avian Influenza Viruses in Mammals

Cheng Zhang [1,2,†], Huan Cui [2,3,†], Chunmao Zhang [2], Kui Zhao [3], Yunyi Kong [2], Ligong Chen [1], Shishan Dong [1], Zhaoliang Chen [1], Jie Pu [2], Lei Zhang [2], Zhendong Guo [2,*] and Juxiang Liu [1,*]

1 College of Veterinary Medicine, Hebei Agricultural University, Baoding 071000, China
2 Changchun Veterinary Research Institute, Chinese Academy of Agriculture Sciences, Changchun 130122, China
3 College of Animal Medicine, Jilin University, Changchun 130062, China
* Correspondence: guozd06@163.com (Z.G.); dkljx@hebau.edu.cn (J.L.)
† These authors contributed equally to this work.

Table S1. Amino acid differences between two H5N6 influenza viruses.

Segment	Position	HB1905	HB1907
HA	44	R	G
	77	S	R
	125	R	S
	128	S	P
	145	A	P
	166	M	I
	173	K	S
	187	S	N
	188	V	A
	193	N	K
	218	A	T
	227	R	S
	531	V	A
NA	26	I	T
	39	V	M
	43	T	A
	46	N	S
	76	V	M
	81	E	G
	130	R	K
	250	K	R
	262	M	I
	266	A	T
	286	R	G
	342	I	T
	345	S	N
	386	D	N
PB2	80	R	K
	137	S	N
	183	M	L
	225	I	V
	332	K	R
	338	I	V
	340	R	K
	348	V	L

451	L	I
457	I	V
461	V	I
490	N	S
508	Q	R
559	N	T
598	T	V
613	A	V
627	E	K
636	M	L
648	L	V
661	S	A
676	T	V
680	N	D
699	R	K
731	A	V

Table S2. Nucleotide similarity of HA gene of two H5N6 isolates.

The Strains in This Study	Similar Strains	Nucleotide Similarity
A/chicken/Hebei/HB1905/2019(H5N6)	A/Jiangsu/1/2018 (H5N6)	99.0%
	A/JiangsuNanjing/1128/2020 (H5N6)	98.6%
A/chicken/Hebei/HB1905/2019(H5N6)	A/duck/Jiangxi/2.28NCNP25K3-OC/2018 (H5N6)	98.8%
	A/Goose/Guangdong/7.20DGCP010-C/2017 (H5N6)	99.3%
